# Carcinoid Tumor of the Cecal Appendix

**DOI:** 10.7759/cureus.30793

**Published:** 2022-10-28

**Authors:** Oscar A Salirrosas Roncal, Christian Tantalean Gutierrez, Cesar Llerena Vasquez

**Affiliations:** 1 Department of Surgery, Hospital Regional Docente de Trujillo, Trujillo, PER; 2 Department of Pathology, Hospital Regional Docente de Trujillo, Trujillo, PER

**Keywords:** pathology, appendectomy, appendicitis, cecal appendix, carcinoid tumor

## Abstract

Cecal appendix tumors are unusual and have a low frequency of presentation, which is reported between 0.2 and 0.5% of all digestive tract tumors. From the mentioned tumors, the carcinoid ones are the most common neoplasms of the cecal appendix and are characterized in most cases by slow growth and an asymptomatic clinical course. However, in some cases, they can present as a metastatic disease with fatal outcomes. We report the case of a 24-year-old female patient with a typical case of acute appendicitis, in whom an additional diagnosis of a carcinoid tumor is obtained upon receipt of the pathology report. The prognosis of appendicular carcinoid tumors is good, with a five-year survival rate of 95%-100% and a recurrence rate of less than 1%.

## Introduction

Acute appendicitis is the most common surgical emergency performed by a general surgeon [1]. It is performed either as definitive management of acute appendicitis or performed prophylactically during an intra-abdominal surgical procedure for another reason [2]. It is estimated that approximately 280,000 appendectomies are performed annually in the United States [3].

Cecal appendix tumors are unusual (which may be due to the small amount of mucosal surface available for malignancy alteration) and have a low frequency of presentation, which is reported between 0.2 and 0.5% of all digestive tract tumors [3]. Regarding demographics, appendicular tumors are more common in women, although this association may, in part, have been attributed to the higher frequency of incidental appendectomies in women undergoing pelvic surgery [4].

Even though primary appendiceal cancers are rare, there is diverse histology. Carcinoids are by far the most common, accounting for approximately 66%, with cyst-adenocarcinoma accounting for 20% and adenocarcinoma accounting for 10%.

Carcinoid tumors originate from the cells of the GI diffuse endocrine system located along the lining of the crypts and in an isolated manner in the lamina propria of the digestive tract [5]. These neoplasms are characterized in most cases by slow growth and an asymptomatic clinical course. However, in some cases, they can present as a metastatic disease with fatal outcomes [6].

Despite its low incidence, its importance lies in the fact that appendiceal tumors are exceptionally diagnosed before or during surgery, the majority being diagnosed through the pathology report of the removed appendix [7].

## Case presentation

A 24-year-old Peruvian woman, previously healthy, presented to the ER with a history of three days of abdominal pain beginning in the mesogastrium that, after 12 hours, migrated to the right iliac fossa. The pain was associated with nausea, vomiting, and hyporexia. She was assessed by the emergency medicine's attending physician, who requested laboratory exams and consults from gynecology and general surgery.

Relevant laboratory results revealed normal hematocrit (37.8%) and leukocytosis (WBC was 11,970 with a WBC differential including 75% of neutrophils, 19% of lymphocytes, and 6% of monocytes).

Gynecology ruled out adnexal pathology. When assessed by general surgery, a diagnosis of acute appendicitis was made, and an appendectomy was scheduled.

A conventional appendectomy was performed without complications: A Rockey-Davis incision was made in the right lower quadrant of the abdomen, and the abdominal wall was dissected through the incision into the peritoneal cavity. After the appendix (with signs of gangrene) was found, the mesoappendix was divided between hemostats and tied with 3-0 silks. The base of the appendix was tied with 2-0 silk, and the appendix was resected using a scalpel. Aponeurosis was closed with running nylon 2-0, and the skin was closed with simple interrupted nylon 4-0. The surgery procedure report stated acute gangrenous appendicitis as the operative diagnosis. The patient progressed favorably and was discharged on postoperative day 1.

**Figure 1:**
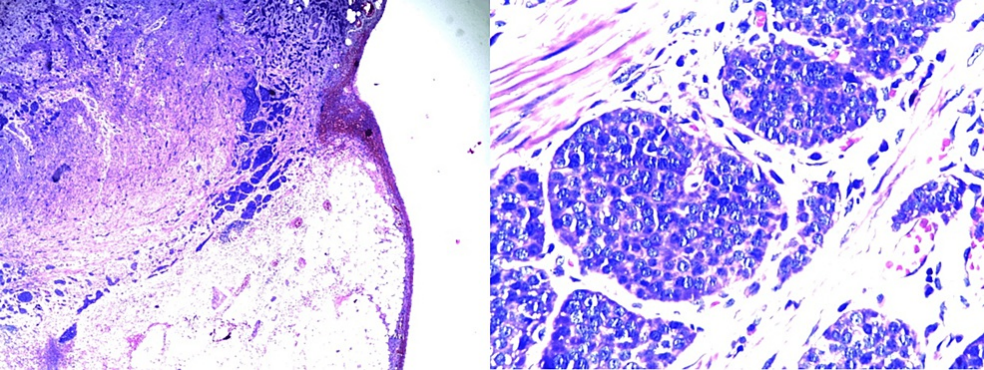
Microscopy of the cecal appendix.

**Figure 2:**
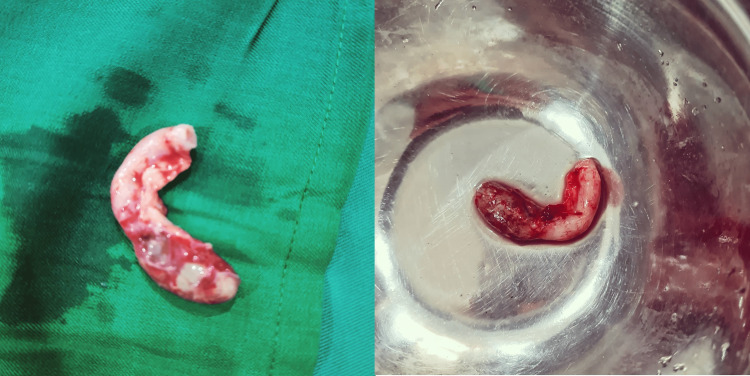
Gross images of the appendix.

The pathology report revealed a well-differentiated carcinoid tumor that infiltrated the serosa, no vascular or lymphatic invasion was observed, and the appendiceal wall presented areas of gangrenous necrosis (Figure 1). On gross examination, the cecal appendix measured 8 x 0.9 x 0.7 cm, showing a dilated lumen occupied by a fecalith (Figure 2). The patient was then transfered to the oncology service for further management.

## Discussion

Carcinoid tumors are well-differentiated neuroendocrine neoplasms that can be found in the GI tract (55%), respiratory tract (30%), and other organs (15%), such as the kidneys or ovaries. In the GI tract, the location in the small intestine corresponds to approximately 45%, in the rectum to 20%, in the cecal suspect to 16%, in the colon to 11%, and in the stomach to 7% [7]. It should be noted that because of the clinical presentation of carcinoid tumors, their incidence is considerably underestimated since many of them are asymptomatic [8,9].

The description made by Lubarsh in 1888 is considered the first to report the characteristics of the carcinoid tumor. Soon after, in 1890, Obendorfert introduced the term carcinoid in an attempt to emphasize its benign nature; therefore, their malignant nature was not given importance until the end of the 1940s, and the possibility of producing metastases from them had been known since 1890 [10].

Carcinoid tumors at the cecal appendix location occur more often between the third and fourth decades of life, affecting women more frequently than men (which corresponds to the case presented) [9]. Its location is usually at the tip of the appendix, and only in some cases, those located in other portions give appendicular symptoms. Carcinoid syndrome (not found in the case presented) is rarely produced by them, and if it is present, it is usually associated with advanced forms of the disease [11]. The histopathological study is vital for the detection of the mentioned tumors. Appendiceal carcinoid tumors appear as yellow or white, firm nodules, most composed of enterochromaffin cells. These serotonin-producing tumors are usually low-grade and contain cells that are argentaffin and argyrophil-positive. The tumors are well-circumscribed and unencapsulated, and consist of tightly packed nests and acini-containing neoplastic cells that fill the mucosa and variably extend into the appendiceal wall. The tumor cells contain abundant amphophilic or eosinophilic granular cytoplasm with round, smooth nuclei and coarse "salt and pepper" chromatin and rare mitoses. In the case described, due to the size and the lack of vascular or lymphatic invasion, it would be classified as stage I according to the TNM classification for carcinoid tumors of the appendix [12]. Treatment of appendiceal carcinoid tumors depends on various factors: tumor diameter, location within the appendix, depth of local infiltration, lymphatic infiltration, presence of metastases, histological type, and patient age; however, definitive therapy is based more on expert consensus than a study with a high level of evidence [13, 14].

In tumors smaller than 1 cm in diameter, the treatment of choice is a simple appendectomy. In comparison, in tumors with a diameter larger than 2 cm, right hemicolectomy with lymph node dissection is recommended [14]. There is controversy about the treatment of tumors between 1 and 2 cm, and it is recommended that it should be individualized. Thus, among the tumors of this last mentioned group, the ones that are located close to the base of the appendix, have vascular or lymphatic invasion of the submucosa, or infiltration of the mesoappendix, it is advisable to perform a right hemicolectomy for young patients, while in patients older than 60 years or with high surgical risk, the recommended treatment is an appendectomy. Additionally, right hemicolectomy is advised in the presence of lymph node metastases, regardless of the patient's age [15]. The prognosis of appendiceal carcinoid tumors is good, with a five-year survival rate of 95%-100% and a recurrence rate of less than 1% [16]. Additionally, besides the tumor diameter and cell type, mitotic cells are other important prognostic factors. In most cases, mitotic cells are seen at less than 1 per 10 high-powered magnification fields. However, if more than 2 or 3 were found per 10 high-powered magnification fields, the prognosis would be very poor [17].

Regarding a differential diagnosis, other types of appendiceal malignancies include cystadenoma, cystadenocarcinoma, goblet cell carcinoid, and colonic-type adenocarcinoma. Mucinous cystadenoma and cystadenocarcinoma are indistinguishable prior to resection when confined to the appendix. They should be resected without rupturing the appendix to reduce the risk of peritoneal seeding and the development of pseudomyxoma peritonei. When suspected preoperatively or encountered intraoperatively, a right hemicolectomy is advised if it is suspected preoperatively or identified intraoperatively. Goblet cell carcinoid and colonic-type adenocarcinoma are more aggressive tumor types. They should be treated similarly to colon cancer, including the right colon resection and adjuvant chemotherapy [18].

Despite all the presented information, its results are contradictory as in some developing countries, the practice of sending all appendices specimens for routine histopathology examination depends on the concerned clinician and is variable. Some authors are against the policy of routine histopathological examination of every appendectomy specimen. They suggest that appendices should be sent for examination only if there is an obvious macroscopic abnormality at surgery [19, 20].

## Conclusions

The finding of an appendiceal carcinoid tumor, and appendiceal tumors in general, is incidental in the vast majority of cases. However, it is a diagnosis that should be considered when facing acute appendicitis, given that the overall survival rate is very high with appropriate and especially timely treatment. Routine pathology study of resected appendices is vital for adequate diagnosis and management. Therefore, we emphasize the importance of obtaining histopathological studies of every removed appendix, given the fact that visual examination does not always correlate with later pathological examination.
